# 

**Published:** 1873-11

**Authors:** 


					﻿LIST OF CASH PAYMENTS TO THE “ RECORD.”
Dr. J. E. Stinson, Texas, 81.25 ; Dr. A. M. Dantzler, Ga., 2.50 ;
Dr. I. L. Case, Tenn., 2.00 ; Dr. J. Q. A. Hudson, Ohio, 2.50 ; Dr.
A- R. Kilpatrick, Texas, 2.50 ; Dr. T. J. Gallaher, Penn., 1.50 ; Drs.
Laxton & Cobbett, N. C., 2.50 ; Dr. E. A. Drewry, Va., 2.50 ; Dr. J.
W. Hill, So. Ca., 2.50 ; Dr. J. J. Grace, Ala., 2.50 ; Dr. R. Fowler,
Miss., 2.50 ; Dr. John O’Brien, N. C., 1.25 ; Dr. W. C. McMillan,
S. C., 2.50 ; Dr. R. F. Wyche, N. C., 2.50 ; Dr. W. L. Best, No. Ca.,
1.25 ; Dr. Wm. Law, Miss., 2.50 ; Dr. E. S. Foster, N. C., 2.50; Dr.
R. S. Tidwell, Tenn., 2.20 ; Dr. J. L. Martin, Oregon, 50c. balance ;
Dr. R. H. Day, La., 2.50 ; Dr. J. W. Dupree, La., 2.50 ; Hon. I. E.
Shumate, Ga., 2.00 ; Dr. H. L. Petrie, Miss., 2.50; Dr. W. W. Farr,
Miss., 2.50 ; Drs. Binford & Dement, Ala., 4.00 ; Dr. W. S. Harris,
Ala., 2.50 ; Dr. L. J. Sherrill, Ala., 2.50 ; Dr. H. T. Bahnson, N. C.,
2.50; Dr. T. C. Morton, Ala., 2.50 ; Dr. E. P. Booth, Texas, 2.50 ;
Dr. A. Bulla, N. C., 2.50 ; Dr. J. G. Thomas, Ga., 2.50 ; Dr. C. M.
Connor, Ga., 2.50; Dr. H. P. Guilbean, La., 2.50; Dr. D. M. McRae,
Miss., 2.50 ; Dr. J. F. Newell, N. C., 2.50 ; Dr. W. D. Hurst, Ky.,
2.50 ; Dr. G. D. Norris, Ala., 2.50 ; Dr. J. W. Quillian, Ga., 2.50 ;
Dr. F. M. Gray, Ala., 2.50 ; Dr. W. S. Selman, Ga., 2.50; Dr. John
F. Carmichael, Ky., 2.50 ; Dr. H. L. Watkins, Ga., 2.50 ; Dr. W. J.
Moore, Va., 2.50; Dr. L. B. Edwards, Va., 2.50; Dr. Thomas E.
Collier, Ga., 2.50; Dr. G. H. Burnett, Tenn., 50 cts. balance; Dr.
A. B. Pierce, N. C., 2.50 ; Dr. J. H. Shelton, N. C., 2.50 ; Dr. Benj.
Rhett, S. C., 50 cts. balance; Dr. F. M. Fitzhugh, Miss., 2.50; Dr.
Neil Gillis, Ga., 2.50; Dr. O. Gregory, N. C., 2.50; Dr. E. T. Henry,
Miss., 2.50; Dr. J. A. Griffin, Ga., 2.00; Dr. L. G. Anderson, Ga.,
2.50; Dr. T. W. Shockley, Ala., 2.50; Dr. H. M. Lanier, Miss., 2.50;
Dr. T. J. Charlton, Ga., 2.50; Dr. J. L. Gibson, Ga., 2.50; Dr. C. D.
Parke, Ala.. 2.50; Dr. C. L. Lamples, Ga., 2.50; Dr. J. G. Pope, Ga.,
2.50 ; Dr. V. G. Hitt, Ga., 2.50 ; Dr. Thomas S. Mitchell, Ga., 2.50 ;
Dr. A. Kingman, Ga., 2.50 ; Dr. T. O. Powell, Ga., 2.50 ; Dr. D. B.
Turner, Miss., 2.50 ; Dr. W. S. Sheppard, S. C., 1.90 ; Dr. J. Harris,
Ga., 2.50; Dr. L. 0. Hicks, Ala., 2.50; Dr. J. B. Mathews, Ga.,
2.50; Dr. Alex. Brown, Ga., 2.50; Surgeon General’s Office, Wash-
ington, D. C., 5.00.
The SouHiprii Medical BeCohL
EDITED BY
T. S. POWELL, M. D.,
AND
W. T. GOLDSMITH, M. I).
ASSOCIATE EDITORS.
GEORGIA.	MISSISSIPPI.	SOUTH CAROLINA.
Robert Battey, M. D.,	E. T. McSwain. M. D.,
R.	C. Word, M. D.,	C. B. Gallaway, M. D.,	G. M. Rivers, AL I).
AV. L. Kirkpatrick, M. D.,	Edward Lea, M. D.,
James A. Long, M. D.,	S. V. D. Hill, M. D.,	m™vrcOT.T,
W. L. M. Harris, AI. D.,	AV. B. Harvey, M. I).,	TENNESSEE.
II. L. Battle. M. D.,	J. W. M. Shattuck, M. D.,	_	m	,	„„
W. C. Musgrove, M. D.,	E. T. Henry, AI. D..	'J-	JP^er,	AL D	.
L. B. Bouchelle, M. D..	T. P. Lockwood, AI. D..	Swan	Burnett, AL	D.
John C. Drake, M. D.,	Robert Kells, M. D.,
E F. Knott, M D	TEXAS.
Thomas Burdell, M. D.,
v	vidotvi.	S. M. Welch, AT. D.,
V	Bitt, M. D.,	AIRGINIA.	T ,j Heard, AL D..
J. E. Pope, M. D.,	w A Heard M D
J A Hunnicutt M D.,	A. R. Kilpatrick, M. D.
A. II. Brantley, M. D..	Charles S. Lewis, M. D..
J. J. Knott, M. D.,	John II Claiborne, M. D.,
J. C. Wisdom, M. D.,	F. Horner. Jr.. AI. D.,	ARKANSAS.
W. W. Leake, M. D.	R. J. Preston, Al. D.,	, TT ,	,,
S Payne M D	A. B. Hodge, M 1)..
NORTH CAROLINA.	J.’e.’ Lockridge. AL D..	J- K- Bodge, M. D.
.1. S. Wharton, M. D.,
J. R. Hughes, M. D.<	Win. (). Owen, AI. D.,	ALABAMA.
S.	S. Satchwell, M. D.,	Sam’l P. Ripley, AL D.,
A.	B. Pierce, AL D.,	Benj. Blackford. AL D.,	J. C. Knox. AL D.,
II. Kelly. AT. I)..	E. A. Drewry. AL D..	Geo. F. Taylor, AL D.,
Geo. A. Foote, M. D.,	Rob. B. Tunstall, Al. D.,	J. P>.	Harnett, AL D.,
E. Afliknderson, AL D.,	AV. F. Barr, AL D..	S. Al.	Hogan. AL D..
II. TYBahnson. M. D.,	L. B. Anderson. AL D.,	D. S.	Hopping, AL D.,
Willis Alston, AL I)..	S. L. Jepson. AL D., W. Va., J. 'I'.	Searcv. AL D.,
Thos. F. Wood. AL D..	J. Al. Lazzell, AL I).	S. F.	Ilill. AL D.
N. J. Pittman, M. D.
CORRESPONDING EDITORS :
Ely Van DeWarker. AL D., New York.	AL II. Ilenr.. Al, D . New A’ork.
John IT. Weir, M. D., Illinois,	Surgeon to N. Y. Dispensary. Dep’t of
Thos. J. Gallaher. AL D., Pennsylvania,	Venerial and Skin Diseases.
Robert Robson, AI. I)., Indiana,	Wm R. Whitehead. AT. 1).. Colorado Territory,
C. C. F. Gay, AL D.. New York,	A. Patton. AI D.. Indiana.
Surgeon of Buffalo General Hospital.	Beniamin Howard, AL I).. New York,
AV. T. Taylor, AL D., Kentucky,	Charles Kearns, AT. D., Kentucky.
B.	W. Stone. AL D., Kentucky.	S. C. Bussey, M. D.. Washingtoil, D. C.,
Assistant Physician Western Lunatic Asylum.	‘A. AV. Hobson, AL D., Arkansas.
James Tyson. AI. I).. Philadelphia. Pa.,	A. W. Calhoun. AL D., now Berlin,	Prussia.
W. AV. Leen, AI. D.. Philadelphia, Pa.,	T. Curtis Smith. AT. D.. Ohio.
Geo. Strawbridge. AL D., Philadelphia. Pa.
All business Letters should be addressed to POWELL & GOLDSMITH,
Post Office Box 527, Atlanta, Ga.
				

